# Patients' preferences regarding shared decision-making in prosthodontic treatment: a university clinic-based cross-sectional study

**DOI:** 10.1038/s41415-025-9300-4

**Published:** 2026-02-13

**Authors:** Franz J. Strauss, Maurice Moser, Aspasia Pachiou, Daniel S. Thoma, Nadja Naenni

**Affiliations:** 41415616820001 Clinic of Reconstructive Dentistry, Center of Dental Medicine, University of Zurich, Zurich, Switzerland; Universidad Autónoma de Chile, Santiago, Chile; 41415616820002 Clinic of Reconstructive Dentistry, Center of Dental Medicine, University of Zurich, Zurich, Switzerland; 41415616820003 Clinic of Reconstructive Dentistry, Center of Dental Medicine, University of Zurich, Zurich, Switzerland; Department of Periodontology, Research Institute for Periodontal Regeneration, Yonsei University College of Dentistry, Seoul, Republic of Korea

## Abstract

**Introduction** Patient involvement in treatment decisions is central to shared decision-making in prosthodontics, yet preferences and perceptions may vary.

**Aims** To evaluate patients' preferred and perceived roles in decision-making related to their prosthodontic treatment.

**Design** Cross-sectional observational study.

**Setting** University-based dental clinic offering prosthodontic care.

**Materials and methods** Patients undergoing prosthodontic treatment completed a questionnaire incorporating the Control Preferences Scale (CPS) to assess their preferred and perceived roles in treatment decision-making. Data were analysed descriptively.

**Results** Most patients preferred a passive (60.9%) or collaborative role (20.3%), with only 1.5% desiring an active role. In contrast, 26.5% perceived themselves as having actively participated in decisions. Patients treated by postgraduate clinicians reported greater active involvement (35.7%) compared to those treated by undergraduates (67.4% reported passive roles). Older patients exhibited a stronger preference for passive involvement.

**Discussion** A mismatch between preferred and perceived roles suggests that patient engagement may evolve during treatment. Clinician experience and patient age significantly influenced perceived involvement, indicating a need for tailored communication strategies.

**Conclusion** Although patients often initially prefer passive roles, involvement can increase with time and clinician expertise. Clinicians should adapt communication approaches to support evolving patient preferences and promote shared decision-making.

## Key points

- Most patients initially preferred a passive role but their engagement often increased over time.
- Patient involvement in shared decision-making varies depending on the clinical setting and the clinician's level of experience.
- Effective shared decision-making requires continuous reassessment of patient preferences and tailored communication to build strong patient-clinician partnerships and support patient-centred outcomes.

## Introduction

Clinical decision-making is a collaborative process between the clinician and the patient, with various models outlining the roles of each stakeholder. In the ‘paternalistic' model, clinicians (viewed as experts) typically recommend their preferred course of action for the patient.^1^ However, this model often fails to consider the patient's desire for autonomy and participation.^2^ Many clinicians argue that current decision-making processes effectively engage patients and provide them with adequate information.^3^ However, patients frequently feel that their involvement in decision-making is limited.^4^^,^^5^

To address these challenges, the collaborative or ‘shared decision-making' (SDM) model promotes equal authority and shared responsibility between the patient and the clinician. This approach positions the patient as an active partner in the decision-making process, with the goal of collectively determining the best possible health outcomes and the way to reach these.^4^ In other words, the objective is to empower patients to choose the treatment option that best aligns with their preferences and needs.

In practice, the SDM process begins with the clinician explaining the medical issue and discussing available treatment options, including the choice of no treatment, while outlining the risks and benefits of each treatment option. The clinician then explores the patient's preferences, addressing their ideas, concerns and expectations regarding the options, while reassuring them that they will not have to make the decision alone but will receive professional support throughout the process. Next, the clinician offers recommendations based on the best available research, their clinical expertise, and the patient's preferences and values. The patient's understanding of the information is assessed and they are encouraged to ask questions. This process should not be rushed and should therefore not occur in a single consultation. For many patients, scheduling a follow-up appointment allows them to have the time to reflect on the discussion and establish a shared decision on the information provided, possibly with input from others.

There are several ways to measure shared SDM processes. To improve the accuracy of SDM measurement, structured and objective tools have been developed. A recent systematic review identified eight instruments designed to assess patients' preferences for SDM, all of which have undergone rigorous psychometric testing.^6^ One of the most widely used tools is the Control Preferences Scale (CPS), which provides a subjective assessment of how involved patients feel in terms of decision-taking/making regarding their healthcare, thus evaluating a key aspect of SDM.^7^

In medicine, there is evidence suggesting that the majority of patients want to be actively involved in their treatment options and often desire more involvement in healthcare decisions than they currently experience.^8^ However, this information is lacking in dentistry, specifically in the field of prosthodontics. To date, little research has explored how patients, clinicians and technicians engage in SDM, how SDM affects satisfaction with treatment outcomes, or how digital technologies may support the process.^9^^,^^10^ The aim of the present study was to assess patients' preferred role in decision-making as well as their perceived role in the decision-making process regarding their planned/received prosthodontic treatment.

## Materials and methods

### Study design and setting

This cross-sectional study was conducted at the Clinic of Reconstructive Dentistry at the Center of Dental Medicine University of Zurich, Switzerland. Data were collected through face-to-face interviews with patients receiving treatment from undergraduate or postgraduate students within the department. The study used the validated CPS^11^^,^^12^ and received ethical approval (Project Nr. Req-2023-00092) in January 2023. Written informed consent was obtained from all participants, and the study followed the principles of the Declaration of Helsinki.

### Participant recruitment

Eligible participants were adults with sufficient proficiency in German who required prosthodontic treatment encompassing fixed or removable prostheses supported by teeth or implants. Inclusion criteria were:

- Age ≥18 years
- Proficiency in German
- Willingness to participate.

Patients were recruited at the Clinic for Reconstructive Dentistry, University of Zurich, between March and June 2024. A single calibrated examiner from the research team approached potential participants during routine dental visits, explained the study purpose and design, and provided additional information on data collection to those who consented. Recruitment was conducted without a standardised script.

### Sample size

A convenience sample of 64 patients was used because the study was exploratory and restricted to those treated at the clinic during the predefined recruitment period. Consequently, no formal sample size calculation was performed. The objective was to generate preliminary data and point estimates to guide sample size determination in future confirmatory trials.

### Data collection

The CPS, originally developed by Degner and Beaton (1987), has been widely used in studies on SDM in dentistry and was deemed appropriate for this research.^11^^,^^12^ The scale consists of five statements representing different decision-making roles: most active, active, collaborative, passive, and most passive (Table 1). All CPS items were provided in German by using the validated German version of the CPS.^13^

**Table 1:** The Five Control Preferences Scale (CPS) statements for assessing patients' role preferences and perceived role

**CPS statement**	**Role**
I prefer to make the decision about which treatment I will receive	Most active
I prefer to make the final decision about my treatment after seriously considering my dentist's opinion	Active
I prefer that my dentist and I share responsibility for deciding which treatment is best for me	Collaborative
I prefer that my dentist makes the final decision about which treatment will be used but seriously considers my opinion	Passive
I prefer to leave all decisions regarding my treatment to my dentist	Most passive

Questionnaires were administered face-to-face by a single trained examiner (MM), following a standard operating procedure and neutral reading of each CPS statement. Patients were initially approached at the start of treatment and again during therapy. Due to difficulties with patient comprehension, the protocol was later revised so that each participant completed the questionnaire only once. Consequently, most patients were surveyed during treatment, with only a minority assessed at the end of therapy. To reduce interviewer bias, interviewers only repeated the item wording and did not provide additional prompts. Participants always completed the preferred-role items before the perceived-role items. On average, completion took 3–5 minutes.

Participants were first asked to select the statement that best described their preferred decision-making role in their treatment. They were then asked to choose the statement that best represented their perceived role in their current treatment (Table 1). This allowed for a comparison between their preferred and actual involvement in decision-making. The CPS was administered either before or during the active treatment phase. All data were initially recorded manually using a clinical report form before being transferred to an Excel spreadsheet. Additional demographic and clinical information, including age and sex, was also documented.

### Statistical analysis

Due to the exploratory nature of this study, responses were analysed using descriptive statistics, with results presented as percentages. Data analysis was conducted using Microsoft Forms, Stata v18, and GraphPad Prism v10.

## Results

**Fig. 1:**
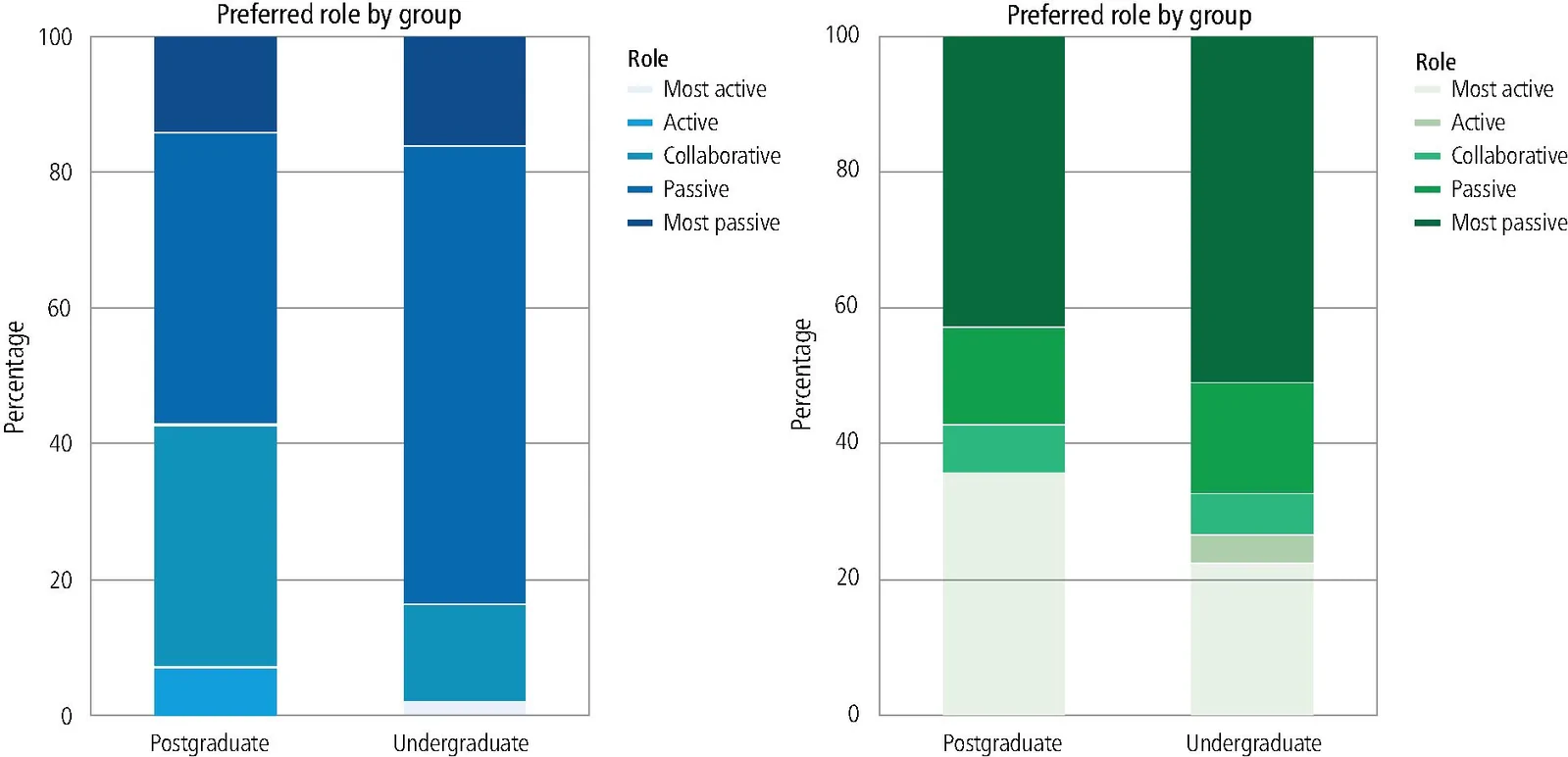
Participants' preferred and perceived roles in decision-making, stratified by the educational level of the treating clinician (undergraduate versus postgraduate)

A total of 64 patients were approached and recruited for the study. The sample had a higher proportion of male patients (76.6%) compared to female patients (23.4%). The mean age was 69 years (standard deviation: 11.3), with an age range of 42–89 years. Most patients were undergoing active treatment. A summary of the participants' preferred and perceived roles in decision-making, stratified by the educational level of the treating clinician (undergraduate versus postgraduate) is presented in Figure 1.

### Preferred role in decision-making

**Fig. 2:**
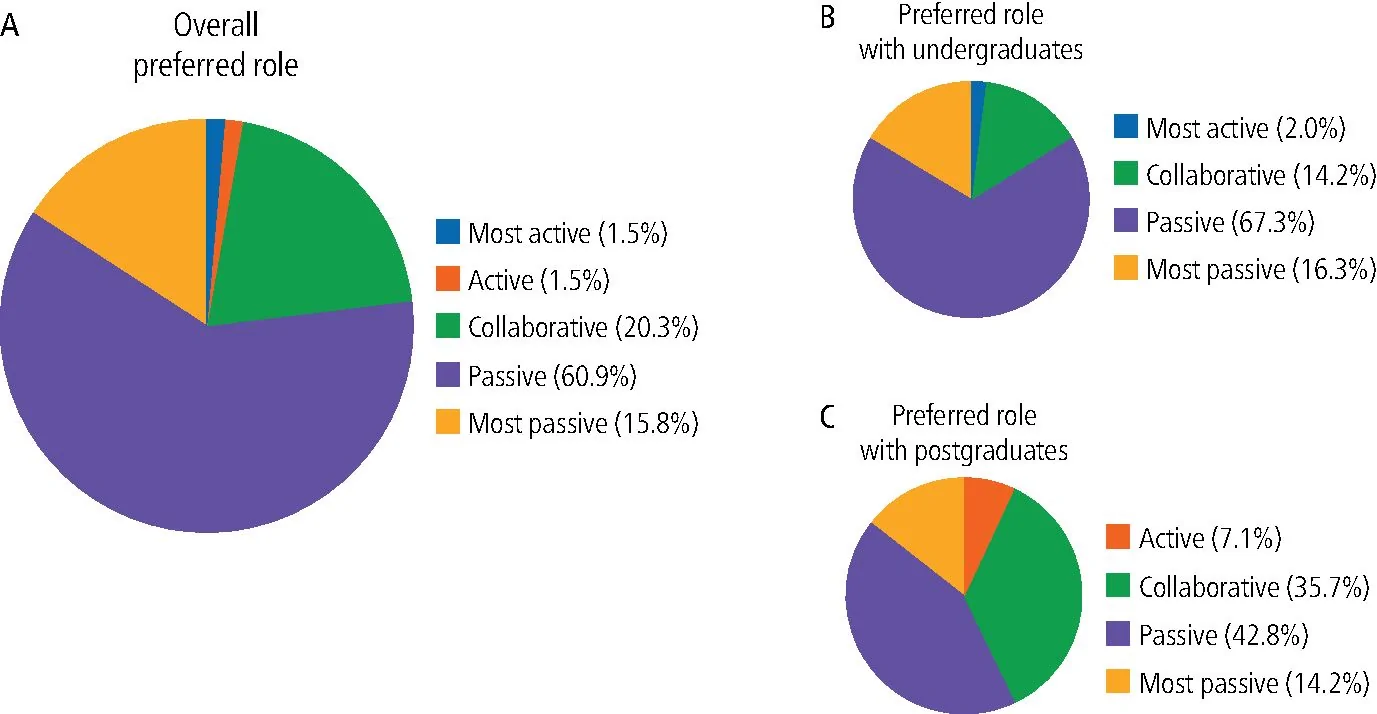
(A, B, C) Preferred roles in treatment decision-making

The majority of patients preferred a passive role (60.9%), followed by a collaborative role (20.3%) (Fig. 2A). A smaller proportion (15.8%) preferred a most passive role, while only 1.5% chose an active (one participant) or most active (one participant) role (Fig. 2A). Among patients treated by undergraduates (n = 49), 67.3% preferred a passive role (Fig. 2B). The next most common choices were most passive (16.3%) and collaborative (14.2%). In contrast, patients treated by postgraduates (n = 14), the most commonly selected role was passive (42.8%), followed by collaborative (35.7%) (Fig. 2C). Fewer patients preferred a most passive (14.2%) or active (7.1%) role (Fig. 2C). Overall, patients treated by postgraduates showed a more evenly distributed preference, whereas patients treated by undergraduates showed a stronger inclination toward a passive role.

### Perceived role in decision-making

**Fig. 3:**
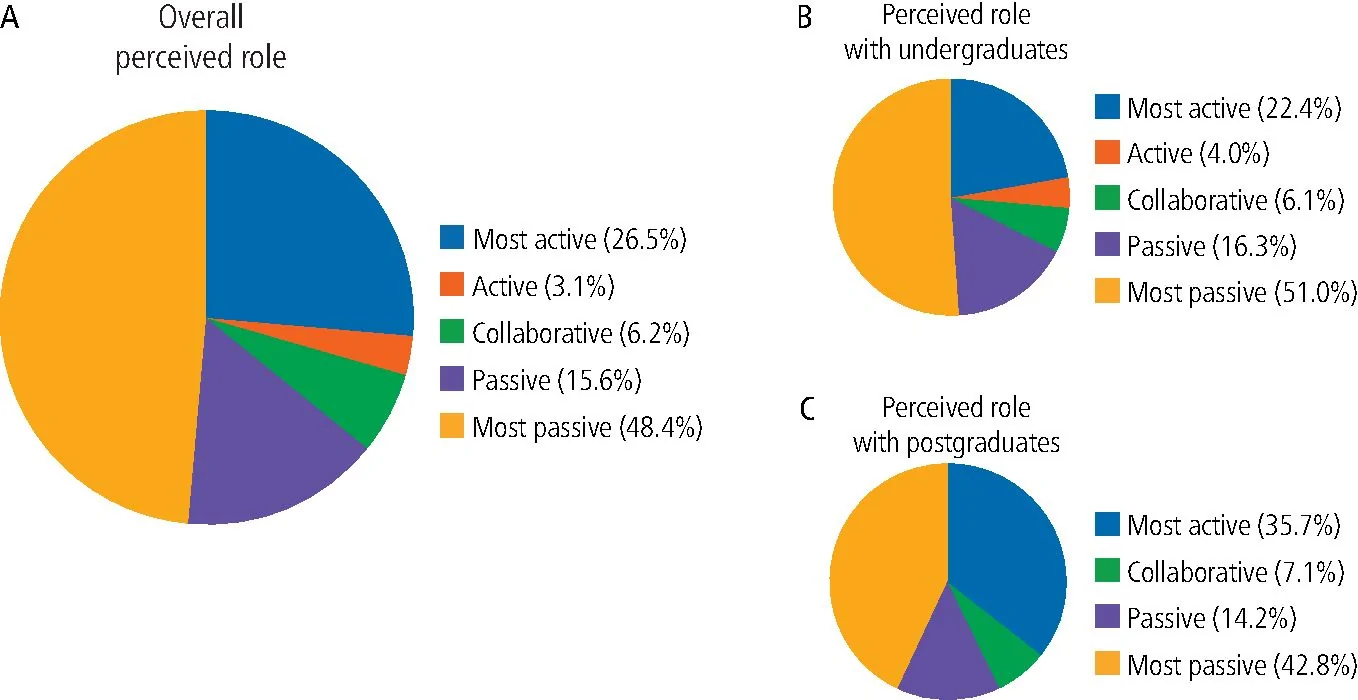
(A, B, C) Perceived roles in treatment decision-making

More than half of the participants perceived their role as most passive (48.4%) or passive (15.6%) (Fig. 3A). Conversely, 26.5% felt their role was most active, while only 3.1% and 6.2% identified their role as active or collaborative, respectively (Fig. 3A). For patients treated by undergraduates, perceptions were more skewed toward passivity, with 51% identifying as most passive (Fig. 3B). The next most common response was most active (22.4%), followed by passive (16.33%), collaborative (6.1%), and active (4%) (Fig. 3B). Among patients treated by postgraduates, role perceptions were more evenly spread (Fig. 3C). The most common response was most passive (42.8%), followed by most active (35.7%), indicating a mix of highly engaged and minimally engaged individuals. Fewer patients saw themselves as passive (14.2%), while an even smaller percentage identified as collaborative (7.1%) (Fig. 3C). Overall, patients treated by postgraduates had a more balanced distribution between most active and most passive while those treated by undergraduates were more likely to perceive themselves as passive or most passive (67.3%).

## Discussion

This study aimed to explore patients' preferred and perceived roles in decision-making related to prosthodontic treatment using the CPS. The results revealed a gap between the level of involvement patients desired and the involvement they actually perceived during treatment decisions. This discrepancy highlights important opportunities to enhance SDM practices within prosthodontics. To date, little research has examined how patients, clinicians and technicians engage in SDM, how SDM influences satisfaction with treatment outcomes, or how digital technologies may support the process.^9^^,^^10^ The present study represents an initial step in addressing these questions within prosthodontics.

The CPS is one of the most widely used instruments for assessing patient involvement in healthcare decisions and has been also used in dentistry.^11^^,^^12^ It offers a valuable framework for evaluating a central aspect of SDM and allows meaningful comparison with previous research. Furthermore, because decision-making inherently incorporates patients' subjective perceptions, the CPS is particularly well-suited to capturing these dynamics.

### Preferred and perceived roles

Most participants expressed a preference for either passive or collaborative roles in decision-making, consistent with findings from previous studies conducted in dentistry.^14^^,^^15^ These studies have indicated that many patients prefer to rely on clinicians' expertise, particularly when faced with complex treatment options. A recent study of 96 patients with Stage III/IV periodontitis examined autonomy and information-seeking preferences. The authors reported a stronger preference for autonomy during initial dental visits than after receiving a diagnosis. They suggested this decline reflects a perceived shift in decision-making responsibility to the clinician, leading patients to rely more on professional expertise.^14^

Conversely, a study of 40 patients in both a hospital and a general dental practice settings in the United Kingdom found that a collaborative role was most preferred, while a passive role was least preferred.^12^ Similarly, a Canadian mail survey of 434 patients regarding the management of teeth with apical periodontitis reported that 44% preferred an active role, 40% a collaborative role, and 16% a passive role.^16^ Differences between these findings and the present study may reflect methodological factors (mail versus in-person surveys), cross-cultural variations,^17^ and differences in healthcare systems.

Interestingly, around one-quarter of our participants reported greater perceived involvement than their initial preferences indicated, suggesting that patient engagement may evolve over time as familiarity and trust in the treatment process increase.^12^ A medical study similarly found that increasing patients' knowledge of available options enhanced their willingness to participate in treatment decisions.^18^ It has been proposed that clinicians who provide longer consultations should be rewarded, as this may promote greater patient participation,^19^ particularly in university settings such as the present one. This underscores the importance of regularly reassessing patient preferences throughout treatment.^15^

### Influence of clinician experience

Patients' perceived involvement varied according to the clinician's level of experience. Those treated by postgraduate clinicians reported feeling more actively engaged, likely due to better-developed communication skills and greater confidence among these clinicians. Previous research has linked clinician experience and communication abilities with increased patient empowerment.^20^ Conversely, patients treated by undergraduate students generally reported feeling less involved, despite frequent appointments, possibly due to the structured educational environment and perceived hierarchy in these settings. The presence of a supervising professor in undergraduate settings may have reduced patients' perceived need to engage actively in SDM. Differences between undergraduate and postgraduate clinics may also reflect the varying complexity of treatment. Undergraduate students must meet curriculum requirements to qualify as general dentists in Switzerland, whereas postgraduate students manage more advanced cases, including prosthetic and surgical procedures within a three-year full-time programme.

### Impact of age

Older participants predominantly preferred passive roles, aligning with existing research indicating that older adults generally desire less active participation in healthcare decisions.^14^ Previous reports suggest older patients might favour clinician-directed decisions due to cognitive limitations, perceived complexity of treatment choices, or traditional views on healthcare professional authority.^14^ However, regardless of their preferred level of involvement, older adults consistently value comprehensive and clear information.^21^ Therefore, age-appropriate and clear communication strategies remain essential to support informed consent and patient understanding, even among those preferring passive involvement.^14^

### Barriers and facilitators to shared decision-making

Although SDM is widely endorsed across healthcare, its consistent implementation in dentistry, particularly in prosthodontics, remains limited.^22^ Several studies have identified persistent barriers, including time constraints, ambiguous definitions and expectations and insufficient training in SDM principles.^23^^,^^24^ Addressing these challenges through structured SDM education and the integration of clear, standardised practices into dental curricula and clinical guidelines could substantially enhance the delivery of patient-centred care.

Qualitative research among general practitioners has also highlighted the risk of overloading patients with information.^25^ Practitioners have expressed concern that excessive or poorly filtered information could cause confusion or fear, potentially discouraging patients from pursuing treatment.^25^ Clinicians therefore face the challenge of providing enough information to ensure informed consent while avoiding overload, a challenge that is especially pressing in hospital settings, where consultation time is often very limited. Since a core goal of SDM is to personalise communication, clinicians must take care to prioritise information that is relevant and meaningful to each individual. Understanding a patient's specific concerns and preferences is key to delivering information that supports, rather than hinders, informed decision-making.

Developing familiarity with a patient over time has been shown to improve the quality and ease of treatment discussions. However, this rapport requires continuity of care, which is often lacking in one-off or emergency appointments. In such settings, the absence of an established relationship can make the implementation of SDM more difficult. Conversely, as observed in the present study, patients treated by more experienced clinicians reported that decision-making felt more collaborative. This suggests that trust and familiarity developed over time can facilitate more effective SDM.

### Clinical implications

Our findings emphasise the necessity for personalised communication that adjusts according to changing patient preferences and perceptions throughout treatment. Implementing structured decision aids^9^ and ensuring repeated patient-centred conversations occur can bridge gaps between preferred and perceived involvement, aligning patient engagement with individual comfort levels and expectations. Dental educators and practitioners should therefore prioritise developing flexible communication skills and promoting environments conducive to appropriate patient engagement.

### Limitations

This study has several limitations. First, it was based on a convenience sample of German-speaking participants, who were predominantly male (76.6%) and skewed toward older adults, which limits the generalisability to broader prosthodontic populations. Second, most patients were surveyed during treatment, with only a minority assessed at the end of therapy, restricting a more comprehensive analysis of changes in patient roles over time. Third, given the exploratory design and the use of a convenience sample, responses were analysed descriptively. Fourth, the complexity of the CPS statements may have affected participants' understanding and responses, underscoring the need for simplified assessment tools and multilingual options in future research. Finally, because the surveys were filled out during clinic visits, stress related to the appointment may have influenced how people responded.

## Conclusions

This study highlights the importance of flexible and responsive SDM approaches in prosthodontic treatment. While patients predominantly prefer passive involvement, perceived engagement often increases over time and with greater clinician experience. Effective SDM necessitates dynamic recognition and response to evolving patient preferences, fostering meaningful patient-clinician partnerships tailored to individual needs.

## Data Availability

The de-identified dataset (CPS responses and basic demographics) and the study questionnaire are available from the corresponding author on reasonable request. Data sharing is subject to the University of Zurich ethics approval (Req-2023-00092), participant consent, and applicable Swiss data-protection regulations; public deposition of raw data were not consented. Aggregated results are reported within the article.
